# Perioperative Radiotherapy in Punch Excision of Trunk Keloids: An Emerging Strategy for Recurrence Control and Aesthetic Optimization

**DOI:** 10.1111/jocd.71003

**Published:** 2026-06-16

**Authors:** Muhammad Talha, Syed Abdullah shah, Ubaid Ur Rehman, Fatima Sohail, Muhammad Hanif Khan

**Affiliations:** ^1^ King Edward Medical University Lahore Pakistan; ^2^ Jinnah Sindh Medical University Karachi Pakistan; ^3^ Spinghar University Kabul Afghanistan

To the Editor,

1

In a recent study entitled “Punched Excision Combined with Radiotherapy for Keloid Treatment” [1] published in the *Journal of Cosmetic Dermatology*, Guan and Huang described the role of punched excision combined with radiotherapy as a therapeutic strategy for the management of keloid. This study provides significant insights into treatment optimization and recurrent management. Keloids represent a challenging fibroproliferative disorder, particularly on the trunk, where mechanical tension exacerbates recurrence and complications after treatment. The surgical excision method leads to high recurrence rates between 50% and 80%, which makes adjuvant radiotherapy (RT) become standard practice to block fibroblast growth and collagen production. The punch excision method uses core punch biopsy to maintain peripheral skin tension, which shows potential for treating trunk keloids, but the best time for administering radiotherapy (both perioperative and postoperative) has not been established because early postoperative complications occur frequently [1].

Postoperative RT establishes a time frame when unregulated inflammation continues during the healing process after surgical tissue removal. The combination of increased cytokine production with myofibroblast activation leads to wound complications resulting in permanent scarring. The risk is especially high for individuals with Fitzpatrick skin types IV through VI because they have a higher tendency to develop hypertrophic scars. Khan et al. reported a 65% recurrence rate with conventional postoperative RT timing disrupting the healing process in darkly pigmented skin. The method treats these problems by directing treatment at the fibroblast activity peak before tissue removal, while punch excision decreases both keloid mass and stem cell activation. The combination treatment systematizes initial inflammatory response, thus decreasing various complications, including hyperpigmentation, wound dehiscence, bleeding, and acute pain while preserving long‐term aesthetic results [2].

The cohort study conducted between 2022 and 2024 tested 34 trunk keloid patients who received punch excision and electron beam radiation therapy, and followed up with the patients for 18 months, showing no recurrence cases, while the POSAS score showed 62.77% improvement from patients and 52.73% improvement from observers. The research demonstrated that Perioperative RT, which started 1 day before the operation, provided better results than postoperative RT, which began within 24 h after the operation, because it resulted in shorter bleeding times and reduced cases of moderate to severe acute discomfort [2].

A recent case series included 30 patients who suffered from refractory multifocal trunk keloids and received treatment through 2‐mm punch excision along with intralesional triamcinolone and single‐fraction electron beam RT occurring within 8 h after their surgical procedure. The study results showed no return of symptoms or worsening of conditions after 12 months, while the participants achieved significant improvements in pain, itchiness, color, stiffness, thickness, and irregularity according to their POSAS scores. The surgical team used this technique during the first hours after surgery to stop patients from developing side effects, including excessive granulation and wound tension, which helped their wounds to heal through secondary intention while their skin remained intact for large areas [3].

The study conducted by Liu et al. in 2026 evaluated the effectiveness of treating medium‐to‐large keloids through bleomycin injection combined with punch excision and X‐ray radiation therapy, resulting in 12.5% of cases returning after 12 months. The authors propose two solutions, including hybrid protocols that combine pre‐excision low‐dose bleomycin treatment with day‐0 electron‐beam radiation therapy and size‐stratified randomized clinical trials to achieve 5% recurrence reduction while minimizing treatment side effects [4].

Socioeconomic barriers create greater difficulties for patients who need access to perioperative RT after punch excision because insurance companies treat keloid treatments as cosmetic procedures. Patients from lower‐income backgrounds develop health conditions at a later stage while facing treatment delays because only 20%–29% of them achieve higher income levels of US$75000 or more. At the same time, people who earn below the poverty line of US$22000 experience continuous cycles of untreated growth, pain and itching and lost work capacity, and financial hardship [5].

The guidelines need to include perioperative radiation therapy as a treatment option for patients with high‐risk trunk keloids because randomized trials have shown its effectiveness in treating both surgical complications and long‐term safety. This method delivers improved results because it functions effectively in environments with different resource availability.

## Author Contributions

Muhammad Talha contributed to conception or design of the work, also accountable for all aspects of the work in ensuring that questions related to the accuracy. Syed Abdullah shah contributed to drafting the work or reviewing it critically for important intellectual content, also responsible for analysis of the data. Ubaid Ur Rehman provided final approval of the content to be published and ensuring compliance with journal guidelines and ethical standards. Fatima Sohail contributed to review of manuscript for clarity and technical accuracy. Muhammad Hanif Khan contributed to review of manuscript for clarity and technical accuracy and provided final approval of the content.

## Funding

The authors have nothing to report.

## Ethics Statement

The authors have nothing to report.

## Conflicts of Interest

The authors declare no conflicts of interest.

## Data Availability

Data sharing not applicable to this article as no datasets were generated or analysed during the current study.
